# The ABNL-MARRO 001 study: a phase 1–2 study of randomly allocated active myeloid target compound combinations in MDS/MPN overlap syndromes

**DOI:** 10.1186/s12885-022-10073-w

**Published:** 2022-09-24

**Authors:** Tamara K. Moyo, Jason H. Mendler, Raphael Itzykson, Ashwin Kishtagari, Eric Solary, Adam C. Seegmiller, Aaron T. Gerds, Gregory D. Ayers, Amy E. Dezern, Aziz Nazha, Peter Valent, Arjan A. van de Loosdrecht, Francesco Onida, Lisa Pleyer, Blanca Xicoy Cirici, Raoul Tibes, Klaus Geissler, Rami S. Komrokji, Jing Zhang, Ulrich Germing, David P. Steensma, Daniel H. Wiseman, Michael Pfeilstöecker, Chiara Elena, Nicholas C. P. Cross, Jean-Jacques Kiladjian, Michael Luebbert, Ruben A. Mesa, Guillermo Montalban-Bravo, Guillermo F. Sanz, Uwe Platzbecker, Mrinal M. Patnaik, Eric Padron, Valeria Santini, Pierre Fenaux, Michael R. Savona, On Behalf of the MDS/MPN International Working Group

**Affiliations:** 1 Vanderbilt University School of Medicine, Vanderbilt-Ingram Cancer Center, 2220 Pierce Avenue, Nashville, TN 777 PRB USA; 2 Levine Cancer Institute, Charlotte, NC USA; 3 James P. Wilmot Cancer Institute, University of Rochester Medical Center, Rochester, NY USA; 4 Paris Diderot University, Paris, France; 5 Institut Gustave Roussy, Université Paris-Saclay, Villejuif, France; 6 Cleveland Clinic, Cleveland, OH USA; 7 Johns Hopkins University, Baltimore, MD USA; 8 Department of Internal Medicine I, Division of Hematology and Hemostaseology, Medical University of Vienna, Vienna, Austria; 9 Ludwig Boltzmann Institute for Hematology and Oncology, Medical University of Vienna, Vienna, Austria; 10 VU University Medical Center, Amsterdam, Netherlands; 11 Fondazione IRCCS Ca’ Granda Ospedale Maggiore Policlinico, University of Milan, Milan, Italy; 12 Third Medical Department With Hematology, Medical Oncology, Rheumatology and Infectiology, Paracelsus Medical University, Salzburg, Austria; 13 Salzburg Cancer Research Institute Center for Clinical Cancer and Immunology Trials, Salzburg, Austria; 14 Institut Català d’Oncologia-Hospital Germans Trias i Pujol, Josep Carreras Leukemia Research Institute, Universitat Autònoma de Barcelona, Bellaterr, Spain; 15 Mayo Clinic, Scottsdale, AZ USA; 16 Sigmund Freud University, Vienna, Austria; 17 H. Lee Moffitt Cancer Center, Tampa, FL USA; 18 University of Wisconsin-Madison, Madison, WI USA; 19 Department of Hematology, Oncology, and Clinical Immunology, University of Duesseldorf, Duesseldorf, Germany; 20 Dana-Farber Cancer Institute, Boston, MA USA; 21 University of Manchester, Manchester, UK; 22 Hanusch Hospital and Ludwig Boltzmann Institute for Hematology and Oncology, Vienna, Austria; 23 University of Pavia, Pavia, Italy; 24 School of Medicine, University of Southampton, Southampton, UK; 25 Université de Paris, APHP, Hôpital Saint-Louis, Centre d’Investigations Cliniques, INSERM CIC 1427, Paris, France; 26 University of Freiburg, Freiburg, Germany; 27 Mays Cancer Center at UT Health San Antonio MD Anderson, San Antonio, TX USA; 28 MD Anderson Cancer Center, Houston, TX USA; 29 Hospital Universitario Y Politécnico La Fe, Valencia, Spain; 30 University Hospital Leipzig, Leipzig, Germany; 31 Mayo Clinic, Rochester, MN USA; 32 MDS UNIT, University of Florence, HematologyFlorence, Italy

**Keywords:** ABNL MARRO, MDS/MPN, ASTX727, Itacitinib, Phase 1b/2

## Abstract

**Background:**

Myelodysplastic/myeloproliferative neoplasms (MDS/MPN) comprise several rare hematologic malignancies with shared concomitant dysplastic and proliferative clinicopathologic features of bone marrow failure and propensity of acute leukemic transformation, and have significant impact on patient quality of life. The only approved disease-modifying therapies for any of the MDS/MPN are DNA methyltransferase inhibitors (DNMTi) for patients with dysplastic CMML, and still, outcomes are generally poor, making this an important area of unmet clinical need. Due to both the rarity and the heterogeneous nature of MDS/MPN, they have been challenging to study in dedicated prospective studies. Thus, refining first-line treatment strategies has been difficult, and optimal salvage treatments following DNMTi failure have also not been rigorously studied. ***ABNL-MARRO*** (**A B**asket study of **N**ove**l** therapy for untreated **M**DS/MPN **a**nd **R**elapsed/**R**efractory **O**verlap Syndromes) is an international cooperation that leverages the expertise of the MDS/MPN International Working Group (IWG) and provides the framework for collaborative studies to advance treatment of MDS/MPN and to explore clinical and pathologic markers of disease severity, prognosis, and treatment response.

**Methods:**

*ABNL MARRO* 001 (AM-001) is an open label, randomly allocated phase 1/2 study that will test novel treatment combinations in MDS/MPNs, beginning with the novel targeted agent itacitinib, a selective JAK1 inhibitor, combined with ASTX727, a fixed dose oral combination of the DNMTi decitabine and the cytidine deaminase inhibitor cedazuridine to improve decitabine bioavailability.

**Discussion:**

Beyond the primary objectives of the study to evaluate the safety and efficacy of novel treatment combinations in MDS/MPN, the study will (i) Establish the *ABNL MARRO* infrastructure for future prospective studies, (ii) Forge innovative scientific research that will improve our understanding of pathogenetic mechanisms of disease, and (iii) Inform the clinical application of diagnostic criteria, risk stratification and prognostication tools, as well as response assessments in this heterogeneous patient population.

**Trial registration:**

This trial was registered with ClinicalTrials.gov on August 19, 2019 (Registration No. NCT04061421).

## Background

Myelodysplastic/Myeloproliferative Neoplasms (MDS/MPN) are a heterogeneous group of clonal myeloid malignancies that harbor features of both myelodysplastic syndromes (MDS) and myeloproliferative neoplasms (MPN). In the adult population, MDS/MPN diagnoses include chronic myelomonocytic leukemia (CMML), atypical *BCR-ABL1* negative chronic myeloid leukemia (aCML), myelodysplastic/myeloproliferative neoplasms with ring sideroblasts and thrombocytosis (MDS/MPN-RS-T), and myelodysplastic/myeloproliferative neoplasms unclassifiable (MDS/MPN-U) [1]. Patients with these disorders can present with hepatosplenomegaly, constitutional symptoms (including weight loss, night sweats, and fevers), and/or consequences of hematopoietic insufficiency (fatigue, infections, and bleeding), as well as thromboembolic events (especially in the MDS/MPN-RS-T group). Bone marrow findings usually demonstrate hypercellularity due to proliferation of one or more myeloid lineages, as well as dysplasia of at least one lineage. Proliferation is often effective in some lineages, with increased numbers of circulating cells that may be morphologically or functionally dysplastic. Simultaneously, other lineages may exhibit ineffective production, so that cytopenias may be present as well.

The World Health Organization (WHO) first classified MDS/MPN overlap syndromes as distinct from both MDS and MPN in 2001 [2]. Prior to the designation of MDS/MPN as a category by the WHO, some cases of MDS/MPN may have been reported as other myeloid neoplasms or may not have been reported at all. Furthermore, the specific diagnoses and features that define the MDS/MPN have been further refined, leading to reclassification of some myeloid neoplasms. As such, understanding the true epidemiology of these rare diseases has been challenging and the frequency likely underestimated. CMML is recognized as the most common of these disorders, with a reported annual incidence of approximately 0.4 cases/100,000/year and represents approximately 70% of MDS/MPN [3, 4].

Patients with MDS/MPN overlap disorders have a highly variable disease course. Some patients live for many years with stable blood counts and few symptoms while others are highly symptomatic and succumb rapidly to their disease. Many require regular transfusion support for disease-associated cytopenias and/or cytoreductive approaches to control leukocytosis or thrombocytosis. All share an increased risk of developing acute myeloid leukemia and are only curable with allogeneic stem cell transplantation. Accepted treatment options, such as cytotoxic chemotherapy and DNMTi, provide small, if any, survival benefits [5–8]. In general, patients with CMML (median survival 20–40 months [9–12]), atypical CML (median survival 12–37 months [13–17]), and MDS/MPN-U (median survival of 22–33 months [16, 18]) have inferior survivals relative to patients with MDS/MPN-RS-T (median survival 76–128 months [19–21]).

### Risk assessment

Of the MDS/MPN overlap disorders, most is known about CMML in terms of factors predicting survival, and several prognostic tools have been validated in CMML. Although CMML itself is clinically a heterogeneous entity and survival times range across a wide spectrum, in virtually all studies, the percentage of blood and bone marrow blasts are the most important factors determining survival [10–12, 22–25]. Individual prognosis in CMML is related to myeloproliferative features (mostly high WBC count, but also splenomegaly, presence of circulating immature cells, and /or elevated serum lactate dehydrogenase (LDH)), cytopenias (thrombocytopenia, anemia, and/or neutropenia) and features of disease progression (peripheral blasts, bone marrow blasts percentage, including promonocytes) [11, 22, 25–27].

Comprehensive genetic studies have scripted the mutational landscape of MDS/MPN. These genetic alterations further refine prognosis, including chromosomal abnormalities [28–30] and gene mutations [22, 31–33]. The latter have recently been included in three distinct prognostic scoring systems [31–33]. Mutations in ASXL1 are included in all three, whereas the molecular CMML-specific prognostic scoring system (CPSS-mol) also includes mutations in *SETBP1*, *NRAS,* and *RUNX1* [31]. A recent study on behalf of the MDS/MPN IWG has validated several prognostic models illustrating comparable performance though significant heterogeneity in prediction outcomes exist among them [34]. These shortcomings could be circumvented by the development of personalized predictions using machine learning on large knowledge databases [35].

Although many prognostic tools have been validated in CMML, only one has been developed for atypical CML [17], and none have been developed for other MDS/MPN subtypes, and the applicability of prognostic tools developed for MDS or CMML has not been fully explored in other MDS/MPN entities. In patients with MDS/MPN-RS-T, younger age and presence of *SF3B1* and/or *JAK2* mutations have been associated with a more favorable outcome [19]. In patients with aCML, age > 65 years, female sex, WBC > 50 × 10^9^/L, thrombocytopenia, and/or hemoglobin < 10 g/dL have been reported to be adverse prognostic findings [13, 14, 17]. As with CMML, both proliferative (high WBC) and dysplastic features have prognostic value in other adult MDS/MPN subtypes [16, 36].

The mutational profile of rare MDS/MPN subtypes seems to partially overlap with CMML, but some characteristic patterns have emerged [16, 37, 38]. MDS/MPN-RS-T is the most distinct entity from a molecular perspective, characterized in most cases by mutations in *SF3B1* in combination with an MPN driver mutation in *JAK2*. Mutations in several other genes may be present, with *ASXL1* and *SETBP1* considered as adverse prognostic markers [36]. *SETBP1* mutations are most commonly seen in aCML, where they are also considered to confer an adverse prognosis [39]. Somatic mutations in *ASXL1*, *TET2* and *SRSF2* are generally common in MDS/MPN, including MDS/MPN-U and, like CMML, having mutations in multiple genes is associated with an adverse prognosis [40, 41].

Incorporation of prognostic features in treatment decisions remains challenging, due to the lack of controlled trials allowing rigorous identification of predictive factors for specific treatments. The CPSS and GFM prognostic scores have been validated in CMML in the setting of DNMTi treatment. In particular, *TET2*mut/*ASXL1*wt patients have higher response rates and prolonged survival with this treatment [42]. A decision analysis akin to those performed in MDS [43] has yet to be performed to identify how prognostic categories could guide the timing of allogeneic stem cell transplantation, which has been restricted to patients with higher-risk disease (eg. CPSS intermediate-2/high risk) based on expert consensus [44, 45]. Stem cell transplant is, however, an option for only a small fraction of patients, given the demographics of this patient population. The AM-001 trial will improve prospective assessment of the models’ heterogeneity on overall outcome in MDS/MPNs.

### Treatment

Current treatment strategies for MDS/MPN overlap syndromes are poorly defined, due in no small part to the overall rarity of these collective diagnoses, as well as their striking clinical and genomic heterogeneity precluding a single “one-size-fits-all” approach.

Importantly, a small number of patients with dysplastic CMML were treated on the initial randomized azacitidine MDS registration studies and demonstrated similar responses compared to the overall MDS population, which led to the FDA approval of azacitidine for this subtype of CMML (i.e. with WBC < 13 × 10^9^ /μL)[46]. Additional Phase II studies have confirmed the efficacy of DNMTi therapy for all subtypes of CMML [47, 48], and thus DNMTi are often considered the de facto standard of care for higher-risk dysplastic CMML and by extension the other MDS/MPN syndromes, particularly MDS/MPN-U, although DNMTi may not alter the mutational allele burden or disease biology [49, 50]. Access to DNMTi therapy for these patients is also (variably) restricted across different healthcare systems. For patients with proliferative CMML, hydroxyurea was more effective and achieved responses faster than cytotoxic chemotherapy with etoposide [8]. Despite this, responses were not complete, and prognosis remained poor. A recently published large (*n* = 949) retrospective cohort study of CMML patients in Austria, patients with higher-risk CMML (myeloproliferative CMML, blasts ≥ 10%, CMML-1/2, or higher-risk CPSS) [51] had significantly improved survival with DNMTi treatment (*n* = 551) versus other treatments (*n* = 398; 20.5 months [95% CI 18.5–23.5] vs 14.3 months [12.2–16.1]; *p* < 0.0001). However, the European multi-center randomized phase III DACOTA trial evaluating decitabine ± hydroxyurea versus hydroxyurea in advanced proliferative CMML (NCT02214407) revealed no difference in outcomes [52]. While the role of DNMTi therapy in proliferatie CMML is still not entirely clear, allogeneic stem cell transplantation remains the only curative treatment option for transplant-eligible CMML patients and should be considered for all higher-risk patients [44].

JAK inhibition has proven effective for a subgroup of CMML patients with constitutional symptoms and splenomegaly [53, 54], and the combination of azacitidine and ruxolitinib for MDS/MPNs have demonstrated safety and encouraging efficacy [55, 56]. Preliminary results of the farnesyl transferase inhibitor tipifarnib in MDS/MPN patients have been encouraging and warrant further investigation [57]. Immunotherapies are also being actively investigated in CMML. The monoclonal antibody tagraxofusp (SL-401) directed against CD123 and conjugated to a truncated diphtheria toxin reported notable reductions in splenomegaly in CMML patients in an interim analysis [58]. Lenzilumab (KB003), a GM-CSF neutralizing monoclonal antibody, has shown promise in preclinical studies and is being actively investigated in early phase clinical trials [59]. Although thrombopoietin receptor agonists have been successfully used in lower-risk MDS subtypes with severe thrombocytopenia [60, 61], eltrombopag elicited meager response rates in an early study in CMML patients with thrombocytopenia and was associated with high risk of developing leukocytosis [62]. Patients with aCML often present with significant leukocytosis and demonstrate an aggressive disease course, for which allogeneic stem cell transplant should be considered. Hydroxyurea is typically recommended for control of leukocytosis. Treatment with DNMTi can be considered, where accessible, particularly in transplant ineligible patients, as well as the use of JAK inhibitors or dasatinib, with the choice of small molecule inhibitors informed by *CSF3R* mutation analysis. Mutations within the juxta-membrane region of *CSF3R* (also known as the granulocyte colony-stimulating factor receptor) dysregulate JAK family kinases and enhance sensitivity to JAK1/2 inhibition, whereas CSF3R truncation mutations lead to activation of SRC family kinases and sensitivity to tyrosine kinase inhibition with dasatinib [63–65].

Patients with MDS/MPN-RS-T, often characterized by *SF3B1* and *JAK2* mutations and with a prognosis considered to be generally favorable to MDS with ring sideroblasts, are often treated with supportive care measures such as erythropoietin-stimulating agents and red cell transfusions for isolated anemia. Due to an increased thrombosis risk, cytoreductive treatment plus low-dose aspirin therapy is often recommended, particularly in the setting of advanced age, JAK2 mutation, or significant cardiovascular disease [36, 66]. Efficacy of lenalidomide in patients with MDS/MPN-RS-T has also been described [67–71], and the potential use of luspatercept or sotatercept in this population is of significant interest [72, 73].

### Key discoveries and unanswered questions

In 2013 and 2014 a consortium of clinical and laboratory experts in MDS/MPN convened in 3 congresses to address topical issues. Culminating from those meetings was the publication of proposed uniform response criteria for MDS/MPN [74]. Until that time, MDS/MPN patients were either excluded from clinical trials or responses were variably determined using criteria developed for other myeloid diseases. The development of uniform response criteria specifically for MDS/MPN would assist with application and translation of clinical trial results of MDS/MPN patients in the real-world setting. Since the first meetings, the MDS/MPN IWG has expanded to include clinicians and researchers at more than 60 institutions across the United States and Europe. The MDS/MPN IWG meets regularly to review evolving data that influences both our understanding of the pathophysiology of MDS/MPN diseases and our approach to treatments. Through these meetings a multitude of fruitful collaborations have already developed to drive the field forward, but ***ABNL-MARRO*** (**A B**asket study of **N**ove**l** therapy for untreated **M**DS/MPN **a**nd **R**elapsed/**R**efractory **O**verlap Syndromes) represents dedication to develop new therapies efficiently for MDS/MPN, and ABNL-MARRO 001 (AM-001) is the inaugural international transatlantic clinical trial. In AM-001, a controlled, thoroughly annotated collection of patient samples will be prospectively established. It will include fresh whole peripheral blood samples to collect plasma, serum, and peripheral blood mononucleated cells (PBMC), and fresh bone marrow aspirate to collect bone marrow mononucleated cells (BMMC) and plasma. Standard assessments for bone marrow core biopsies and aspirates will include morphology, flow cytometry, and karyotype. Fresh samples will be shipped to one of 2 central laboratories located in either the US or Europe. These samples will be used to address key questions in MDS/MPN and will potentially allow identification of key biological differences between the different subtypes.

It is now well established that the mutational landscape of MDS/MPN, which could be preceded by age-related clonal hematopoiesis of indeterminate potential (CHIP) [75], combines a small number of common somatic mutations in DNA methylation, histone modifier and splicing genes with disease-segregated mutations in signaling genes, i.e., RAS pathway mutations in proliferative CMML; *JAK2*, *MPL* and *CALR* mutations in MDS/MPN-RS-T; and *CSF3R* and RAS pathway mutations in aCML [76]. Mutations in *SF3B1* are prognostically favorable, whereas those in *SETBP1* and *ASXL1* consistently predict shorter survival [32, 77]. Mapping of clonal architecture in CMML identified early clonal dominance, intra-tumor heterogeneity in the hematopoietic stem and progenitor cell (HSPC) compartment in which mutations accumulate mostly linearly, and growth advantage to the most mutated cells as characteristic disease features [78].

Epigenetic dysregulation in MDS/MPN involves abnormal histone marking caused by inactivation of chromatin modifiers or abnormal DNA methylation resulting from mutations in DNA methyltransferases and/or *TET2* methylcytosine dioxygenase [76]. DNMTi can restore a balanced hematopoiesis without necessarily decreasing mutation allele burden in circulating myeloid cells, arguing for a role of epigenetic alterations in disease expression and outcome [50]. Analysis of DNA methylation profiles at diagnosis could generate an epigenetic classifier that predicts response to a demethylating drug [79]. *ASXL1* mutations may predict a lower, whereas the *TET2*mut/*ASXL1*wt genotype may herald a higher rate of response to DNMTi [42]. Molecular analyses of neoplastic cells collected in AM-001 may extend genomic investigations to noncoding DNA regions, gene expression and mRNA splicing analyzed by RNA sequencing, and epigenetic changes that could affect and predict the response to tested drugs.

Experimental models developed in mice suggested that, in myeloproliferative neoplasms, feedback loops between mature and immature cells of the clone affect the behavior of HSPCs, either residual healthy cells or clonal cells that propagate the disease and contribute to its installation and development [80, 81]. It could be useful to further explore the role of mature cells of the leukemic clone in MDS/MPN. These cells can be detected and quantified by multiparameter flow cytometry in bone marrow, e.g., plasmacytoid dendritic cells [82], or in the peripheral blood (e.g., monocytes [83, 84]). Analysis of peripheral blood immature granulocytes and T cell subsets could provide additional information on disease response to treatments and patient outcome.

Annotation of inflammatory cytokines in plasma or serum was recently demonstrated to classify CMML patients into three groups with distinct clinical and genetic features and suggested that a decreased plasma level in IL-10 correlated with poor overall survival, even when adjusted for other prognostic features including *ASXL1* mutation [85]. Deregulated cytokines could play a key role in disease expression and suggest innovative therapeutic approaches [86, 87]. To this end, cytokine levels will be measured in PB and BM plasma samples collected from patients included in AM-001 in order to search for mutation-independent predictors of response to treatment. We will deploy global approaches to analyzing the plasma proteome, with potential for novel biomarker discovery beyond what has been done before, and beyond the limitations of a custom panel of the 'expected' potential biomarkers, with potential for novel discovery.

Testing distinct small molecules in AM-001 will provide opportunities to study the respectively targeted pathways for their pathogenic roles in MDS/MPN.

## Study rationale and objectives

Response rates to single agent hypomethylating agents in MDS/MPN are underwhelming, but alternative options are lacking. Furthermore, there are no approved therapies for patients who relapse after or who fail to respond to DNMTi therapy. Both the rarity and heterogeneity of MDS/MPNs have hindered development of therapies for these diseases. Leveraging the expertise and cooperativity of the MDS/MPN IWG research consortium, AM-001 will not only explore novel treatment strategies for MDS/MPN but will also establish the overarching *ABNL-MARRO* framework for future collaborative clinical and correlative investigations to further explore clinicopathologic markers of disease severity, prognosis and treatment response. Centralized pathology and biospecimen management systems will allow for correlative studies to be conducted in order to advance the scientific understanding of disease pathogenesis and responses to treatment. The international collaborative nature of *ABNL-MARRO* will allow a coordinated approach to both the clinical care of MDS/MPN patients and the correlative science, whereby different labs will contribute from their respective strengths, pooling expertise and resources, working together towards a common goal rather than competing in isolation.

### Novel agents under investigation in *ABNL MARRO*-001

The ABNL-MARRO 001 study (NCT04061421) endeavors to evaluate the safety and efficacy of oral combination therapies in MDS/MPN patients. DNMTi such as azacitidine and decitabine inhibit DNA methylation and allow previously silenced genes to be expressed and to exert direct cytotoxic effects on abnormal hematopoietic cells in the bone marrow. Although responses to single agent DNMTi are limited, DNMTi have been employed in various myeloid diseases where more effective treatment options are lacking, including in CMML, in MDS, and in elderly/unfit patients with AML [46, 88–90]. The administration of azacitidine or decitabine is cumbersome for patients, as they are administered daily by subcutaneous or intravenous routes for 5–7 consecutive days each 4-week cycle until disease progression. For patients who do not live-in close proximity to a referral center, the logistics of this therapy can pose a significant hardship, not to mention the inconvenience of infusion times and the common local effects of large volume subcutaneous administrations. ASTX727 is a novel oral formulation of decitabine together in a fixed dose combination with the cytidine deaminase inhibitor cedazuridine which reduces first pass metabolism of decitabine in the gut and liver. The fixed dose combination (FDC) ASTX727 pill containing 35 mg decitabine and 100 mg cedazuridine administered daily on days 1–5 of each 28-day cycle elicited pharmacokinetic/dynamic properties similar to intravenous decitabine dosed at 20 mg/m2 daily × 5 days in subjects with MDS [91], and a phase III study of ASTX727 in MDS and CMML revealed pharmacoequivalence between the oral and IV forms of decitabine when oral decitabine is dosed with cedazuridine. Likewise, the responses to ASTX727 were similar, or exceeded what was seen in earlier clinical trials and expected with parenteral decitabine [92]. This ASTX727 FDC 35 mg/100 mg pill will constitute a DNMTi treatment backbone within AM-001. The first novel targeted agent included in AM-001 is itacitinib, a selective inhibitor of JAK1 signaling which mediates pro-growth and pro-inflammatory responses that may drive proliferation of myeloid cells in the bone marrow. JAK1 signaling may contribute to common presenting symptoms such as fatigue, constitutional symptoms, and weight loss related to cancer-cachexia. Previously, itacitinib has been shown to reduce myelofibrosis symptom burden and spleen size without significant reductions in hemoglobin or platelets [93]. Additional arms will include other small molecule inhibitors (to be determined) that have shown promise in their development in myeloid diseases.

### Study objectives

The primary objectives for the study are to characterize the dose limiting toxicities of novel oral targeted agents in combination with oral ASTX727 (Phase 1) and to test whether the overall response to each therapy warrants further investigation in more definitive trials in MDS/MPN patients (Phase 2). Overall response will include subjects who achieve best response of complete remission (CR), partial remission (PR), optimal or partial marrow response (MR), or clinical benefit (CB) as defined by the MDS/MPN IWG proposed response criteria. The study will evaluate morphologic bone marrow responses and effects of each treatment on patient survival as secondary objectives. Exploratory objectives (Table 1) are also planned to bolster scientific understanding of disease pathogenesis and to evaluate methods used for prognostication and assessment of response to therapy.

**Table 1:** Exploratory objectives of ABNL-MARRO 001

**Exploratory Objectives of ABNL-MARRO 001**
• To investigate genetic biomarkers of response in MDS/MPN
• To characterize molecular responses to individual treatments
• To evaluate synergistic effects of hypomethylation by ASTX727 and specific pathway blockade by study compounds
• To explore the use of automated quantification of spleen volume from CT exams as a measure of clinical benefit
• To test and/or validate diagnostic algorithms and prognostic indices for MDS/MPN patients
• To investigate the correlation of patient reported outcomes with disease severity and/or treatment response

## Study design

AM-001 is an open label, phase 1b-2 study that will randomly allocate between novel treatment arms in MDS/MPN.

- Arm 1: ASTX727 + itacitinib (INCB039110; JAK1 inhibitor)
- Arm 2: To be determined
- Arm 3: To be determined

**Fig. 1:**
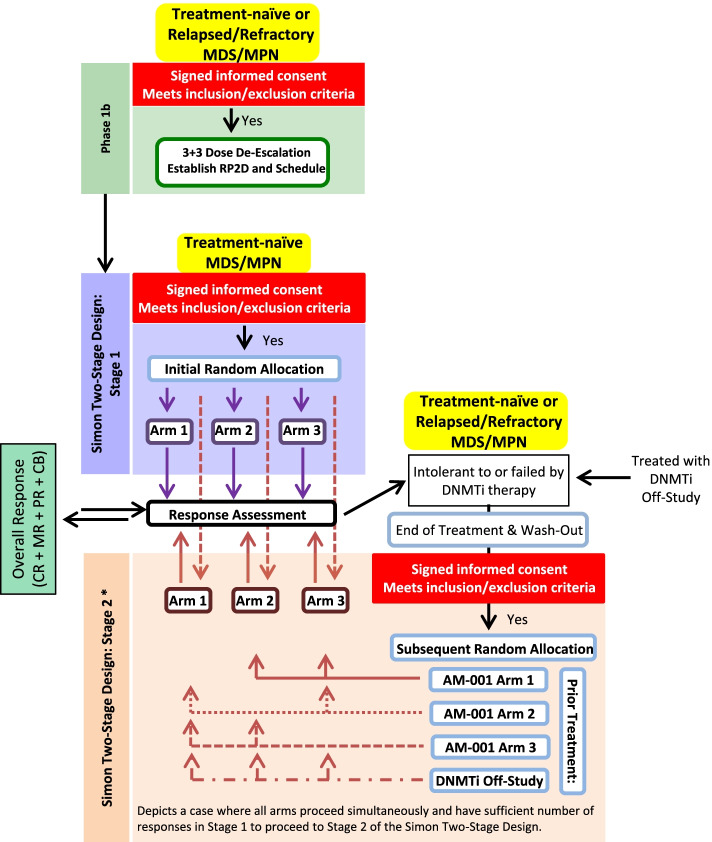
Study Design. Once the RP2D and schedule has been determined for a given treatment in the phase 1b, that treatment arm may enter phase 2, which will follow a Simon Two-Stage design. Stage 1 of the phase 2 will include treatment-naïve MDS/MPN patients only. If sufficient efficacy is demonstrated in treatment-naïve patients to proceed to Stage 2 of the phase 2, then patients who have failed or were intolerant to DNMTi-containing regimens, including treatment on other AM-001 arms or prior to enrolling in the study, will also be included. Eligible patients will be randomly allocated to AM-001 arms that are actively accruing and to which they have not had prior exposure. In Stage 2, patients will be stratified based on treatment status (e.g. treatment-naïve vs relapsed/refractory/intolerant)

The Phase 1b study will allow for the determination of the recommended phase 2 dose and schedule of combination by dose de-escalation. Treatments found to be safe in the phase 1b, or single therapies, will advance directly to the phase 2 portion of the study. The phase 2 study (Fig. 1) will follow a Simon Two-Stage design to determine if there is sufficient efficacy to warrant further investigation of the treatment combination in larger studies for MDS/MPN patients. The arms are not intended to compete against ASTX727 alone or with one another. Rather, the safety and efficacy of each treatment combination will be evaluated independently, and random allocation will serve to guarantee that the cohorts enroll equally.

### Study sites and patient selection

The AM-001 study is the first clinical study to be conducted within the framework of the *ABNL-MARRO* in conjunction with the MDS/MPN IWG. Eligible patients (Table 2) will be enrolled at selected MDS/MPN IWG member institutions across the US and Europe. Adult patients must have a morphologically confirmed diagnosis of MDS/MPN based on the 2016 WHO diagnostic criteria, including CMML, aCML, MDS/MPN-RS-T and MDS/MPN-U.

**Table 2:** Eligibility criteria for ABNL-MARRO 001

**Inclusion Criteria**
• At least 18 years of age and willing and able to meet all study requirements
• Morphologically confirmed diagnosis of MDS/MPN, excluding juvenile myelomonocytic leukemia, according to the WHO (2016) diagnostic criteria
• Treatment-naïve patients with no prior disease-modifying therapy may enroll in any AM-001 arm that is open to accrual in phase1b or phase 2
• After an appropriate wash-out period, patients who have failed or were intolerant to prior therapy regimens containing a DNMTi may enroll in any arm accruing in phase 1b or in the second stage of the phase 2
• Willing to undergo bone marrow biopsy with aspiration and tissue collection for disease assessment and correlative studies during screening and periodically throughout the trial
• Eastern Cooperative Oncology Group (ECOG) performance status of ≤ 2
• Life expectancy of at least 3 months
• For previously treated patients, recovery to Grade ≤ 1 or baseline of any toxicities due to prior systemic treatments, excluding alopecia
• Adequate hepatic and renal function during screening
**Exclusion Criteria**
• Patients will be excluded from arms that contain novel targeted agents to which they have prior exposure, with exception of ASTX727
• Prior receipt of any investigational therapy within 30 days or 5 half-lives before receiving the first dose of AM-001 study therapy
• Prior receipt of any systemic non-investigational antineoplastic therapy, excluding hydroxyurea, within 21 days or 5 half-lives before receiving the first dose of AM-001 therapy
• Known hypersensitivity to decitabine
• Transformation to acute myeloid leukemia
• Organ transplant recipients including allogeneic hematopoietic stem cell transplant
• History of clinically significant or uncontrolled cardiac disease
• History of abnormal EKG or presence of abnormal screening EKG that is clinically significant and contraindicated for clinical study
• Known contraindications to use of ASTX727
• Active and clinically significant bleeding
• Other active malignancy, excluding non-melanoma skin cancer, cervical carcinoma in situ, breast carcinoma in situ, or localized prostate cancer controlled with hormone therapy
• Receipt of wide-field radiotherapy within 28 days or limited-field radiation within 14 days prior to initiating AM-001 treatment
• Patients who require continuation of a prohibited concomitant medication
• Active, uncontrolled infection
• Major surgery requiring general anesthesia within 4 weeks prior to starting AM-001 therapy (other than placement/removal of vascular access devices)
• Women who are pregnant or lactating
• Women/men who expect to conceive/father children within the projected study period and/or who are unwilling to use highly effective methods of contraception throughout the study duration
• Any concurrent serious or unstable medical or psychiatric condition that would jeopardize the patient’s ability to provide informed consent or to comply with the protocol
• Any psychological, familial, geographical or sociological condition that would jeopardize the patient’s ability to comply with the study protocol

### Phase 1b safety run-in

Each combination therapy will be tested first in a safety run-in (Phase 1b) to determine the recommended phase 2 dose (RP2D) and schedule using a 3 + 3 de-escalation design. The initial dose of ASTX727 will consist of the FDC of oral DAC/cedazuridine (35 mg/100 mg) that has been found to approximate the area under the curve (AUC) of standard dosing of DAC [91]. The starting dose of itacitinib in the initial safety cohort has been extrapolated from phase 1 testing when combined with standard DNMTi in other myeloid diseases. Each combination therapy will be allowed a maximum of four dose decrements in the dose de-escalation design. The RP2D and schedule of each combination therapy will be defined as the highest dose and schedule at which no more than 1 of 6 treated subjects experienced a dose-limiting toxicity (DLT). Any treatment arm in which more than 1 of 6 subjects experience a DLT after 4 dose decrements will be terminated. The safety run-in will include both treatment-naïve and relapsed/refractory MDS/MPN patients assigned to a treatment arm based on slot availability.

### Phase 2

For doses that elicited fewer than 2 DLTs in 6 MDS/MPN subjects, efficacy will then be evaluated in phase 2 using a Simon Two-Stage design to allow early discontinuation of any futile treatment regimen and to pursue potentially beneficial combinations in larger cohorts of patients. Eligible subjects will be randomly allocated into an active, non-blinded treatment arm. Treatment will begin at the RP2D and schedule of each combination determined in phase 1b. Stage 1 of the Two-Stage design will include only treatment-naïve subjects, and no stratification will occur prior to randomization. If sufficient responses (including MDS/MPN IWG response categories of CR, PR, MR, or CB) are seen in the first stage in treatment-naïve subjects to warrant further investigation, MDS/MPN patients who are refractory to or who have relapsed after previous DNMTi therapy will also be included in the second stage, and subjects will be stratified in the second stage based on treatment status (treatment-naïve versus relapsed/refractory) prior to randomization (Fig. 1, Table 2).

Response to treatment will be assessed after 2 and 6 cycles of therapy (e.g., on Cycle 3 Day 1 and Cycle 7 Day 1) by physical examination, hematologic laboratory parameters, bone marrow biopsy and/or aspiration, computed tomography (CT) of the abdomen, and patient reported outcomes according to the modified proposed MDS/MPN IWG response criteria. Subjects who have definitive disease progression after 2 cycles of therapy or who experience disease progression after an initial response will discontinue treatment within that arm but may be re-randomized to another AM-001 arm (if available) that has proceeded to the second stage of the phase 2. All patients enrolled in the study will continue therapy until progression of disease, unacceptable toxicity, revocation of consent, or failure to fulfill reasonable study requirements.

### Statistical plan

For the phase 1b, each dose cohort will include a minimum of 3 subjects. The first cohort of 3 patients in an AM-001 arm will be enrolled at the starting dose and schedule of ASTX727 and the novel targeted agent as defined in the protocol, and no additional cohort will be enrolled until the previous cohort has been fully evaluated for toxicity. The DLT evaluation period will be defined as the first 28 days after initiation of treatment on any AM-001 arm in the phase 1. If ≤ 1 of 3 subjects in the first cohort experiences a DLT, the cohort will be expanded by an additional 3 patients treated with the same dose/schedule of each drug. If ≤ 1 of 6 subjects experience a DLT, the RP2D and schedule has been determined. If > 1 subject in either the first 3 or 6 subjects treated at any dose level experiences a DLT, the maximum tolerated dose (MTD) has been exceeded, and the dose(s) of either or both drug(s) will be de-escalated in the next cohort of 3 subjects based on planned dose level reductions defined in the protocol. Dose de-escalation will continue until the RP2D and schedule is determined or until a maximum of 4 dose de-escalations has occurred on that arm. Any arm in which more than 1 of 6 subjects experience a DLT after 4 dose decrements will be terminated and that treatment combination will be deemed too toxic for further evaluation in phase 2. The minimum sample size for the phase 1b evaluation of each treatment arm is 6 patients, and the maximum number of patients in the phase 1 evaluation of each treatment arm is 30 (6 subjects × 5 dose levels).

In phase 2, each arm will follow an optimal Simon Two-Stage design [94]. Criteria for the Simon Two-Stage design for each arm are based on the results of studies of ASTX727 and other DNMTi therapy in myeloid diseases. Accordingly, the null hypothesis that the overall response rate (combined CR + MR + PR + CB) is 35% will be tested against a one-sided alternative. In the first stage, 14 treatment-naïve patients will be accrued in each AM-001 arm and will receive treatment at the RP2D and schedule of the novel targeted agent and of ASTX727, as determined in the phase 1b. If there are 13 or fewer responses in these first 35 patients before the 7^th^ cycle of therapy, the study will be stopped for futility. Otherwise, 52 additional patients (including both treatment-naïve subjects and subjects relapsed after/refractory to other DNMTI-containing therapies) will be accrued for a total of 87. The null hypothesis that the true response rate is 35% or less will be rejected if 38 (44%) or more responses are observed in 87 patients before the 7^th^ cycle of therapy. This design yields a type I error rate of 0.05 and power of 85% when the true response rate is 50%. Assuming low (35% RR) efficacy, the probability of early termination and expected sample size of any arm is 68% and 51.8 patients, respectively. The responses of subjects treated in the phase 1 of an arm will not be included in the determination of futility or efficacy in phase 2. The actual number of patients treated per arm will be dependent on the number of responses obtained in the minimum number of patients treated. If sufficient responses have been observed after 6 cycles of therapy (e.g., by Cycle 7 Day 1 response assessment) in subjects enrolled in stage 1 and criteria of the Simon Two-Stage design are met, the arm will begin enrolling both treatment-naïve and previously treated MDS/MPN subjects in stage 2 of the phase 2. If sufficient responses are observed in the maximum number of subjects enrolled in phase 2 by the second scheduled response assessment (e.g., Cycle 7 Day 1), the null hypothesis will be rejected, and definitive trials may be warranted. If insufficient responses are observed after all subjects in either stage have completed six cycles of therapy, the arm will be terminated.

### Study oversight and guidance

Multiple committees and subcommittees have been organized for the development, oversight, and management of AM-001 and for analysis of AM-001 study data. The Protocol Development Committee has drafted and refined the protocol based on input of all other committee members and will continue to refine the protocol if amendments are needed. The Patient-Reported Outcomes (PRO) and Symptom Assessment Committee advises on the collection, use and analysis of data collected directly from the study subjects regarding their personal study experience (including but not limited to symptom scores and quality of life metrics). The Correlative Science and Biospecimen Committee oversees the collection and utilization of biological specimens collected from each subject in conjunction with AM-001. The Risk Assessment and Criteria Validation Committee advises on the collection and analysis of data that may be used to devise and/or test prognostication indices and to validate the MDS/MPN IWG response criteria. The Operations Committee assists with all operational aspects of the study from study inception to completion. All committees will report to the Executive Committee, which oversees all aspects of the trial, and is led by the Global Study Chair. The study has a Medical Monitor and an independent Data Safety Monitoring Board (DSMB) to ensure the protection of subjects enrolled in the study. The DSMB will meet regularly and as needed if patient safety issues arise, will report their findings to the Executive Committee with recommendations on study continuation, and may propose amendments to the study protocol as necessary.

## Selected study procedures

Subjects will be treated according to the study calendar, barring any adverse events that may warrant dose interruption or modification. All protocol-indicated treatments in this study are orally self-administered, although some doses will be required to be self-administered under direct observation of the study staff. Self-administration of protocol-indicated medications will be recorded in a pill diary which will be reviewed to assess compliance with study medications.

### Safety analysis

After signing the informed consent, adverse events will be collected as detailed in the schedule of assessments. Adverse events will be assessed from the time of consent until at least 30 days after a patient’s last dose on trial or until the subject initiates anti-neoplastic treatment that is not part of AM-001 – whichever occurs first. Safety data will be reported with summary statistics.

### Response assessments

Response assessments will occur after 2 and 6 cycles of therapy and at the end of treatment (EOT). Response assessments will include assessment of spleen size and volume by physical examination and CT, respectively; evaluation of hematologic laboratory parameters, patient-reported symptoms, and bone marrow aspiration and/or biopsy. Responses to treatment will be measured according to the proposed MDS/MPN IWG response criteria [74]. Since these response criteria have not yet been validated in a large independent study, post hoc analyses are planned to determine the validity and clinical utility of these criteria across MDS/MPN subtypes.

### Patient-reported outcomes

Patients with MDS/MPN overlap syndromes may present with a myriad of symptoms that affect quality of life. The MDS/MPN IWG response criteria have incorporated the myeloproliferative neoplasm symptom assessment form total symptom score (MPN-SAF/TSS) which has been rigorously validated in myelofibrosis. There are currently no patient-centered metrics that have been designed specifically for MDS/MPN or validated in a mixed population of MDS/MPN diagnoses. AM-001 will provide opportunity to explore the applicability of the MPN-SAF across these heterogeneous disease groups and to explore alternative patient-centered metrics, including the EORTC-QLQ C30 and the MDS QUALMS, to further understand the impact of disease-related symptoms and how each treatment may affect quality of life.

### Central pathology

Diagnostic criteria for MDS/MPN are now well established and distinguish this group of diseases from either MDS or MPN [1, 95]. Despite this, the diagnosis of these diseases can be challenging for a number of reasons. First, the diagnosis requires both dysplastic and proliferative features, but some cases carry these features to different degrees. A prime example is CMML, for which diversity in presentation has been well established. Some cases present with more prominent dysplastic features and less monocyte proliferation, and others present with more prominent monocytic proliferation and less dysplasia [23, 96]. Additionally, clearly diagnosed myelodysplastic syndrome or myeloproliferative neoplasms can show features that suggest overlap disorders. For example, both primary myelofibrosis and polycythemia vera can show monocytic or neutrophilic progression, making them difficult to distinguish from CMML or aCML [97–99]. Finally, MDS/MPN must be differentiated from benign proliferations, such as reactive monocytosis.

Accordingly, central pathology review in AM-001 presents an opportunity to address some of these diagnostic difficulties. Entry into the trial will not depend on central review, but rather on diagnosis at the local site. However, central pathology review will be performed post hoc with a goal of verifying local diagnosis and to catalog and evaluate any differences between local diagnosis and expert review. Furthermore, a detailed central pathology review will allow evaluation and refinement of the morphologic component of the response criteria [74].

### Prognostic indices

A “global” MDS/MPN prognostic index that has been validated across MDS/MPN subtypes and that informs treatment in MDS/MPN remains elusive. Demographic, clinical and pathologic data will be collected from subjects enrolled in AM-001 to test the applicability of available prognostic indices across MDS/MPN subtypes. Molecular data will also be collected to explore the impact of emerging genetic risk factors in these rare and heterogeneous diseases that may not have been incorporated into indices that were developed prior to widespread use of genomic analysis.

### Research correlates

In addition to pathologic specimens for response assessments, additional bone marrow aspirate and peripheral blood specimens will be collected in screening prior to initiation of AM-001 therapy, with each response assessment, and at the EOT. These specimens will be processed by two centralized laboratories (one in USA and one in EU) and will be used for planned studies to further explore the relationships between the genetic landscape, the variable expression of specified genes/proteins, plasma biomarkers, and patient outcomes (Table 1). The distribution and use of samples by MDS/MPN IWG members are subject to availability and approval by the AM-001 Executive Board and the Correlative Science and Biospecimen Committee.

## Discussion

*ABNL MARRO* is an innovative international cooperative study group designed to address several key challenges in MDS/MPN that have long hindered the development of optimal treatments. By leveraging the MDS/MPN IWG member sites, AM 001 will meet a clear unmet medical need and will enroll both treatment-naïve MDS/MPN patients of all subtypes and MDS/MPN patients who have relapsed after or who are refractory to DNMTi. Although the study is built to add additional arms in real time, the randomization into arms is only to allow equal allocation, and not designed to measure treatments against one another. Rather, the arms will be independently evaluated for both safety (phase 1b) and efficacy (phase 2) in this patient population. If safe and effective, this study could lead to further randomized studies, and ultimately, an expanded armamentarium of treatment options available to both treatment-naïve and relapsed/refractory MDS/MPN patients. Importantly, we aim to explore all oral combination therapies, which could expand both access to and palatability of treatment options for patients who currently must travel for daily infusions for up to one out of every 4 weeks indefinitely and who may endure local site reactions with subcutaneous administration of DNMTi or face problems with venous access.

The data collected from this study will provide robust insight into future treatment strategies, with exploration of risk factors and markers of disease prognosis across MDS/MPN subtypes that may help to guide treatment selection and/or timing. The collection of biospecimens for both planned exploratory research and potential future studies will further inform our understanding of mechanisms of disease, which can then be used to further hone the treatment of these rare and heterogeneous diseases.

Perhaps most importantly, AM-001 is the inaugural study that has established the infrastructure and collaborative network for future prospective interventional studies to advance treatment and to explore clinical and pathologic markers of disease severity, prognosis and treatment response under the umbrella of the *ABNL MARRO*, an clinical trial platform for the MDS/MPN International Working Group.

## Data Availability

Not applicable.

## Abbreviations

ABNL MARRO: A Basket study of Novel therapy for untreated MDS/MPN and Relapsed/Refractory Overlap Syndromes
aCML: Atypical BCR-ABL1 negative chronic myeloid leukemia
AM-001: ABNL-MARRO 001 study
AUC: Area under the curve
AZA: Azacitidine
BM: Bone marrow
BMMC: Bone marrow mononucleated cells
CB: Clinical benefit
CHIP: Clonal hematopoiesis of indeterminate potential
CMML: Chronic myelomonocytic leukemia
CPSS: CMML-specific prognostic scoring system
CPSS-mol: Molecular CMML-specific prognostic scoring system
CR: Complete remission
CT: Computed tomography
DAC: Decitabine
DLT: Dose-limiting toxicity
DNMTi: DNA methyltransferase inhibitor(s)
DSMB: Data safety monitoring board
EOT: End of treatment
EU: European Union
FDC: Fixed dose combination
GFM: Groupe Francophone des Myelodysplasies
HSPC: Hematopoietic stem and progenitor cell
IWG: International Working Group
LDH: Lactate dehydrogenase
MDS: Myelodysplastic syndromes
MDS/MPN: Myelodysplastic/myeloproliferative neoplasms
MDS/MPN-RS-T: MDS/MPN with ring sideroblasts and thrombocytosis
MDS/MPN-U: MDS/MPN unclassifiable
MPN: Myeloproliferative neoplasms
MPN-SAF/TSS: MPN symptom assessment form total symptom score
MR: Optimal or partial marrow response
MTD: Maximum tolerated dose
PB: Peripheral blood
PBMC: Peripheral blood mononucleated cells
PIM: Proviral integration site of Moloney murine leukemia virus
PR: Partial remission
PRO: Patient-reported outcomes
RP2D: Recommended phase 2 dose
USA: United States of America
WBC: White blood cell count
WHO: World Health Organization
